# Detection of known gene fusions in cancer cell lines using whole-genome bisulfite sequencing data

**DOI:** 10.1038/s41598-026-40803-0

**Published:** 2026-03-12

**Authors:** Taehoon Kim, Duhee Bang

**Affiliations:** Department of Chemistry, Yonsei University, 50 Yonsei-ro, Seodaemun-gu, Seoul, 03722 Korea

**Keywords:** Whole-genome bisulfite sequencing, Gene fusion, Split read alignment, Cancer cell lines, BCR-ABL1, Biological techniques, Biotechnology, Cancer, Computational biology and bioinformatics, Genetics, Molecular biology

## Abstract

Whole-genome bisulfite sequencing (WGBS) provides multiple molecular information layers including methylation, copy number variations (CNVs), single nucleotide variants (SNVs), and fragmentome patterns in cell-free DNA (cfDNA) while gene fusion detection from WGBS data has not been widely explored. We applied a custom analysis pipeline using bisulfite-aware aligners supporting split read alignment to detect known gene fusions from WGBS data and validated this approach using cancer cell lines with characterized fusion events. Using K562 cells harboring the *BCR-ABL1* fusion, WGBS-derived breakpoints showed concordance with WGS-derived coordinates. Serial dilution experiments with K562 DNA in NA12878 background at average 63X coverage established a limit of detection (LoD) of 8.1% fusion-positive DNA fraction, demonstrating 100% detection rate at ≥10%. Furthermore, our method with MCF-7 cells detected 10 of 12 validated fusions with high technical reproducibility (Pearson *r* > 0.99), including both inter-chromosomal and intra-chromosomal events. This approach confirms that known gene fusions can be reliably detected from WGBS data, particularly well-suited for applications where methylation and fusion profiling are both required.

**Supplementary Information:**

The online version contains supplementary material available at 10.1038/s41598-026-40803-0.

## Introduction

Gene fusions are structural variants resulting from genomic rearrangements that join two distinct genes and are prevalent in specific cancer types^1,2^. These rearrangements can lead to upregulation^3,4^, loss-of-function^5,6^, or the generation of chimeric proteins^7,8^, all of which can induce tumorigenesis. Notably, specific gene fusions function as molecular classifiers that define clinically distinct cancer subtypes^9–11^. This classification has direct implications for treatment selection, as exemplified by *BCR-ABL1* in chronic myeloid leukemia (CML) and *TMPRSS2-ERG* in prostate cancer^12–14^. Therefore, identifying validated fusion events provides significant insights into cancer subtype determination within diagnostic frameworks.

Beyond fusion events, DNA methylation alterations represent another critical hallmark of cancer^15–17^. These alterations include global hypomethylation of the epigenome and hypermethylation of CpG island promoter regions^18,19^. Recent advances in cancer detection have increasingly relied on multi-feature approaches that integrate diverse molecular signals^20–24^. In this context, whole-genome bisulfite sequencing (WGBS) has emerged as a particularly powerful platform, as it captures multiple information layers from a single assay: genome-wide base-resolution methylation patterns, copy number variations (CNVs), single nucleotide variants (SNVs), and fragmentomic signatures in cell-free DNA (cfDNA). This multi-layered data generation makes WGBS especially valuable for liquid biopsy applications, where maximizing information extraction from limited samples is critical.

Although WGBS provides comprehensive molecular profiles, gene fusion detection has been notably absent from this repertoire, with fusion analysis predominantly relying on alternative sequencing methods such as RNA-sequencing (RNA-seq) or whole-genome sequencing (WGS)^25–28^. This limitation arises primarily from the dual technical challenges posed by bisulfite conversion in WGBS. First, bisulfite treatment causes DNA fragmentation, making it difficult to preserve intact genomic information^29,30^. Second, the conversion of unmethylated cytosines to uracil (subsequently read as thymine) reduces sequence diversity and complicates the accurate alignment required for identifying structural rearrangements^31–33^. While tools such as DELLY have been benchmarked on WGBS data for CNV detection^34^, targeted approaches for identifying gene-level fusions from WGBS data have not been developed.

To address this gap, we evaluated the fusion detection performance in WGBS data from cell lines. Our analysis revealed that fusion breakpoints identified by WGBS in K562 cells were concordant with those detected by WGS. Serial dilution experiments with K562 and NA12878 demonstrated that the limit of detection (LoD) of WGBS-based fusion call was 8.1% at an average sequencing depth of 63X. Additionally, we assessed the capacity of this approach to identify multiple concurrent fusion events using MCF-7 WGBS data, detecting 10 of 12 validated fusions with high reproducibility (Pearson *r* > 0.99).

## Results

### An overview of WGBS-based fusion detection

**Fig. 1:**
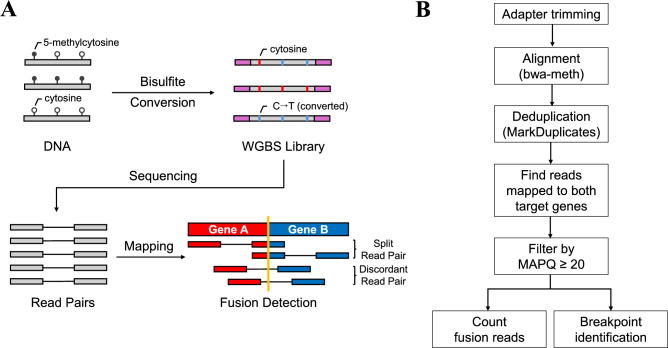
Scheme for WGBS-based fusion detection method. (**A**) WGBS-based fusion detection relies on identifying specific types of read pairs spanning fusion breakpoints. We defined split read pairs as read pairs where one read contains split-alignment spanning the fusion breakpoint with the mate read mapping to the other target gene involved in the fusion. Discordant read pairs are defined as read pairs mapped to different target genes. Read pairs with unmapped reads are excluded from both categories. (**B**) The bioinformatics pipeline utilizes bwa-meth, which supports split-alignment essential for fusion detection. Following adapter trimming and alignment, deduplication is performed using MarkDuplicates to obtain unique read pairs. Read pairs mapping to both target genes are filtered using SAMtools with a MAPQ threshold ≥ 20. Fusion breakpoints are identified by analyzing supplementary alignment positions, which represent boundaries within primary alignments. The pipeline quantifies both split and discordant read pairs supporting fusions and determines breakpoint coordinates.

WGBS data consists of sequenced reads derived from bisulfite-treated DNA fragments, which sacrifices alignment rate to gain methylation information^35^. This hindrance caused by reduced sequence diversity limits the reliable detection of structural rearrangements such as gene fusions in WGBS. Given this limitation, we applied an alignment-based fusion detection method to investigate the feasibility of identifying fusion breakpoints in WGBS data (Fig. 1A and Fig. 1B). This approach is enabled by bisulfite-aware aligner that supports split read alignment. Split read pairs and discordant read pairs were analyzed as evidence for fusion events, where split read pairs directly span breakpoints and discordant read pairs provide indirect support by mapping to both target genes^36,37^.

### Validation of fusion detection using K562 WGBS

**Fig. 2:**
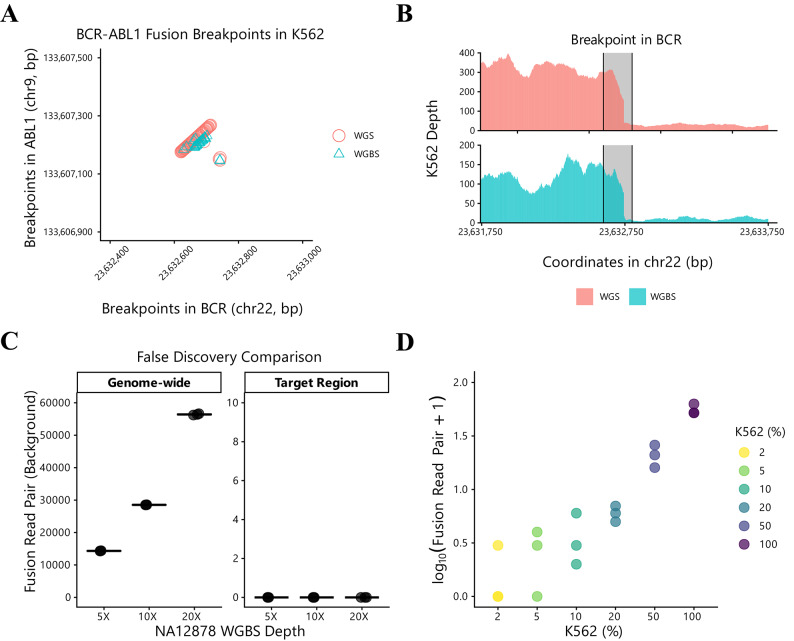
Single fusion detection with K562 WGBS. (**A**) Comparison of unique *BCR-ABL1* fusion breakpoints detected by WGBS and WGS. Each point represents unique breakpoint coordinates simultaneously present in both *BCR* and *ABL1* genes (circles: WGS; triangles: WGBS). (**B**) Coverage profiles of the *BCR* gene surrounding the *BCR-ABL1* fusion breakpoint. Gray shaded areas indicate the coordinate ranges where *BCR-ABL1* breakpoints were detected (top: WGS; bottom: WGBS). (**C**) Assessment of the number of fusion read pairs within different genomic regions in NA12878 WGBS (*n* = 3). "Genome-wide" refers to fusion read pairs detected outside the target region (*BCR-ABL1*), while “Target Region” refers to fusion read pairs detected within the target region. All samples showed zero counts in the target region across different sampling depths (5×, 10×, 20×). (**D**) Number of fusion read pairs supporting *BCR-ABL1* detection across different K562 fractions in NA12878 DNA from the serial dilution experiment (*n* = 3; Spearman *ρ* = 0.926, *p* < 0.001).

K562 cell line is derived from CML and contains the well-characterized cancer-associated fusion *BCR-ABL1*^38^. As a proof of concept, we validated the potential of WGBS for fusion detection using this cell line. Breakpoints in *BCR* and *ABL1* were plotted and compared between WGS and WGBS data (Fig. 2A, Fig. S1). The breakpoints identified in both sequencing methods clustered together in 2D space, demonstrating that WGBS can detect fusion events with similar accuracy to WGS. Previous studies have reported localized coverage drops near fusion breakpoint regions^39–41^. To further support evidence for the fusion event, we evaluated coverage depth at *BCR-ABL1* breakpoints (Fig. 2B, Fig. S2) and found that coverage simultaneously dropped in both WGS and WGBS data, indicating the presence of the *BCR-ABL1* fusion. Additionally, we examined methylation patterns near the fusion breakpoint region, demonstrating the feasibility of concurrent epigenetic profiling alongside fusion detection in WGBS data (Fig. S3).

Downsampling of K562 WGBS reads showed that unique fusion read pairs increased linearly with sampled coverage (Fig. S4), demonstrating the coverage-dependent nature of target fusion detection. Given this depth-dependent increase in target signals, we next examined background fusion read pairs in a fusion-negative control to assess whether increasing coverage also elevates background noise in WGBS. Using NA12878 WGBS data, fusion read pairs were evaluated separately in the whole-genome space and in the *BCR-ABL1* target region (Fig. 2C). In the whole genome, false-positive fusion read pairs were detected even at low coverage (5×) and increased proportionally with downsampled coverage. In contrast, no fusion read pairs were detected in the *BCR-ABL1* target region across all depths, indicating that background noise remains effectively suppressed when analysis is restricted to the target region. A similar trend was observed in WGS data (Fig. S5), supporting the feasibility of WGBS-based targeted fusion detection even under lower mapping quality and higher false positive conditions (Fig. S6).

To determine the detection threshold for WGBS-based fusion detection, we sequenced K562 and NA12878 mixtures at various percentages ranging from 2% to 100% (Fig. 2D). An average coverage of 63X was used to ensure robust detection across the dilution series tested. Detection rates were 33.3% for 2% K562, 66.6% for 5% K562, and 100% for 10% to 100% K562 samples. Using NA12878 WGBS data as negative controls, LoD was calculated as 8.1%. These results demonstrate that WGBS can reliably detect *BCR-ABL1* and may be applicable to other fusion detection purposes when fusions are consistently present and appropriate sequence coverage is achieved.

### Expansion of WGBS to multiple fusion detection in MCF-7

**Fig. 3:**
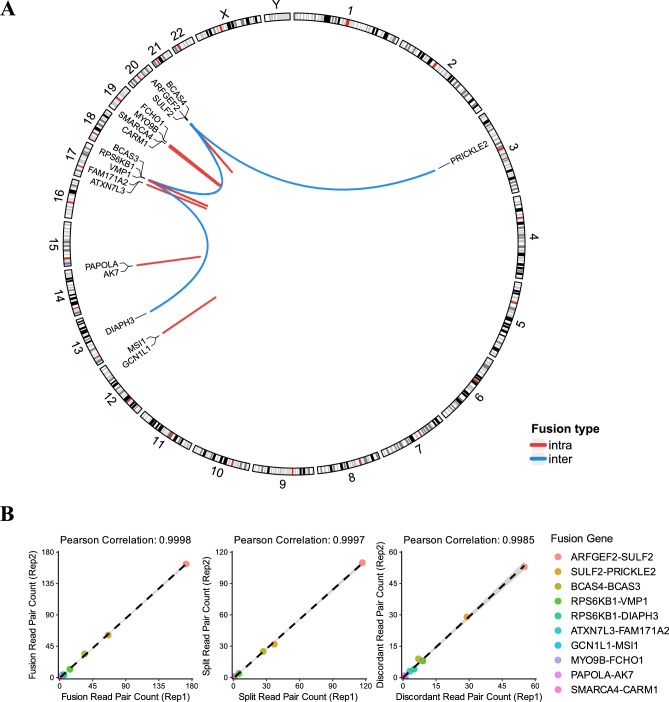
Multiple fusion detection achieved by MCF-7 WGBS. (**A**) Circos plot for the fusions detected in MCF-7 WGBS. Each link represents fusion events, and the color indicates to the type of fusion event (red: intra-chromosomal; blue: inter-chromosomal). (**B**) Replicate concordance of fusion read pair counts (Pearson *r* > 0.99, *p* < 0.001). Read pairs are categorized by total fusion read pairs (left), split read pairs (middle), and discordant read pairs (right).

To evaluate whether WGBS can detect multiple fusion events, we analyzed MCF-7 WGBS data. Among 12 experimentally validated gene fusion events reported in MCF-7^42^, our method detected 10 fusions (Fig. 3A, Table S1). The detected fusions included both inter-chromosomal and intra-chromosomal events, demonstrating the broad applicability of our approach. Both split read pairs and discordant read pairs showed high concordance within replicates (Pearson *r* > 0.99). These fusion events appear to be cell-line-specific, with limited evidence of recurrent observation in breast cancer patients^43^. Notably, comparison of fusion read pair counts revealed that false-positive fusion signals detected across the whole genome greatly outnumber true-positive signals observed in the target regions (Fig. S7). This contrast indicates that WGBS is more suitable for targeted fusion detection, where background noise can be effectively controlled, rather than for genome-wide de novo fusion discovery.

## Discussion

In this study, we demonstrated that WGBS data can be repurposed for gene fusion detection, addressing a notable gap in the analytical potential of this widely-used sequencing platform. By leveraging bisulfite-aware aligners that support split read alignment, we successfully identified fusion events in cell lines containing single fusion (K562) and multiple fusions (MCF-7). The concordance between WGBS-derived and WGS-derived fusion breakpoints in K562 cells validates the technical feasibility of our approach (Fig. 2A, Fig. S1). Importantly, our analysis of background noise in fusion-negative samples (NA12878) revealed that while genome-wide false-positive fusion read pairs are substantially elevated in WGBS data, target regions show zero background signal across all sequencing depths (Fig. 2C, Fig. S5). These findings demonstrates that bisulfite conversion, despite reducing sequence complexity, does not fundamentally compromise targeted fusion detection. Our findings establish that WGBS can provide fusion information alongside its primary methylation profiling capability, enabling comprehensive multi-omics analysis that includes methylation, CNV and SNV from a single assay.

Several limitations must be acknowledged. First, our validation was limited to cell line data with well-characterized fusion events. Cell-line-derived datasets represent homogeneous populations with uniform fusion sequences that may not reflect the biological heterogeneity of primary cancer samples. Critically, fusion-positive cancers often exhibit patient-specific and intra-tumoral breakpoint variations that cannot be represented by uniform cell line models^44–47^. While K562 harbors the clinically validated *BCR-ABL1* fusion that may share sequence characteristics with patient-derived CML samples, it cannot capture the breakpoint heterogeneity observed across different patients or within individual tumors. Furthermore, most MCF-7 fusions have not been recurrently observed in primary breast cancer^43^. Therefore, the clinical utility of WGBS-based fusion detection remains to be validated using patient-derived samples, including both tissue biopsies and liquid biopsies from cancer patients. Second, direct comparison between WGS and WGBS is limited due to different protocols. WGS does not involve bisulfite conversion, naturally resulting in more intact molecules without bisulfite-induced fragmentation. Therefore, our aim was to qualitatively assess WGBS potential for fusion detection rather than provide quantitative platform comparison. Third, the fusion detection performance achieved in WGBS presents practical limitations for clinical implementations. Our demonstrated LoD of 8.1% fusion-positive DNA fraction is well-suited for tissue biopsy scenarios where tumor content is typically high, but may be insufficient for liquid biopsy applications where circulating tumor DNA (ctDNA) fractions are often below 1%. Furthermore, achieving 63X coverage would incur substantial sequencing costs, limiting the practical feasibility of WGBS-based fusion detection in routine clinical workflows. Fourth, two known fusions in MCF-7 (*AC099850.1-VMP1* and *ADAMTS19-SLC27A6*) were not detected by our pipeline. While reduced sequence complexity from bisulfite conversion likely contributes to this limitation, another explanation is that the breakpoints may occur outside our gene reference boundaries. Our approach requires that both fusion breakpoints fall within annotated gene regions, and fusions with breakpoints in intergenic regions or distant from gene annotations would not be captured. This highlights an inherent limitation of gene-focused fusion detection strategies and suggests that future refinements could benefit from expanding the genomic search windows around genes of interest.

For prospective clinical applications, three major areas require further investigation. First, validation using multiple cell lines with diverse fusion events and clinical samples with matched orthogonal fusion detection methods, such as RNA-seq, is essential to establish the sensitivity and specificity required for practical implementation. Second, sequencing coverage should be optimized to achieve a balance between detection sensitivity and cost efficiency. Third, a systematic comparison of computational approaches for fusion detection in WGBS data is necessary to identify best practices and define the most effective pipelines. Evaluating diverse bisulfite-aware aligners and fusion callers will improve analytical robustness and reproducibility.

## Conclusion

We demonstrated that WGBS data, a widely-used platform for methylation profiling, can be repurposed for gene fusion detection. This capability reveals an untapped analytical potential of WGBS, allowing comprehensive molecular characterization from a single assay. Although further validation using clinical samples is required, our findings indicate that WGBS-based fusion detection could enhance multi-feature cancer detection strategies by providing comprehensive molecular information from a single assay. As the use of WGBS continues to grow in both research and clinical contexts, the fusion information embedded within these datasets represents a valuable yet underexplored resource that merits systematic investigation.

## Materials and methods

### Source of genomic DNA (gDNA)

K562 cell line was obtained from the Korean Cell Line Bank and maintained in RPMI 1640 (Gibco, USA) supplemented with 10% fetal bovine serum and 1% penicillin-streptomycin. gDNA was extracted using the DNeasy Blood & Tissue Kit (QIAGEN). Purified NA12878 gDNA was purchased from the Coriell Institute.

### WGBS library preparation

gDNA was sheared to approximately 300 bp using M220 Focused-ultrasonicator (Covaris). Sheared K562 cell line DNA was mixed with sheared NA12878 DNA at varying ratios (2%, 5%, 10%, 20%, 50%, and 100% K562). Spike-in controls consisting of pUC19 (0.1%) and lambda DNA (0.1%) from the NEBNext Enzymatic Methyl-seq Kit (New England Biolabs) were added to the sheared DNA.

End repair, A-tailing, and adapter ligation were performed using the NEBNext Enzymatic Methyl-seq Kit (NEB). Bisulfite conversion was then conducted using the EZ-96 DNA Methylation-Direct™ MagPrep Kit (Zymo Research), with denaturation at 98 °C for 8 minutes and conversion at 64 °C for 3.5 hours. All other steps followed the manufacturer’s protocol.

Post-conversion libraries were amplified by PCR in 50 µL reactions containing 25 µL of NEBNext Q5U Master Mix (NEB) and 5 µL of index primers (10 µM). Thermal cycling conditions were as follows: initial denaturation at 98 °C for 30 seconds; 7 cycles of 98 °C for 10 seconds, 62 °C for 30 seconds, and 65 °C for 60 seconds; and final extension at 65 °C for 5 minutes. Final libraries were quantified using D1000 ScreenTape (Agilent). Sequencing was performed on an Illumina NovaSeq 6000 platform, with each sample receiving an average of 1.3 billion reads.

### Sequencing data processing

Sequenced reads were trimmed using FASTP (ver. 0.20.1)^48^ and mapped to the hg19 human reference genome supplemented with lambda and pUC19 sequences using bwa-meth (ver. 0.2.7)^49^ with the –do-not-penalize-chimeras option. Duplicates were removed with Picard MarkDuplicates (ver. 4.0.5.1)^50^. Methylation calling in CpG, CHG, and CHH contexts was performed using MethylDackel (ver. 0.3.0)^51^. Bisulfite conversion rates were assessed using spike-in controls. For lambda DNA, the ratio of converted cytosines across all contexts was calculated. For pUC19, the preservation of expected CpG methylation was evaluated. Whole-genome conversion rates in the CHH context were also determined (Table S2).

### Fusion call

To identify fusion gene candidates, we extracted reads mapping to both target gene loci from aligned BAM files. Read pairs were filtered to retain only primary alignments (SAMtools^52^ ver. 1.10; excluding secondary and supplementary alignment entries) where both mates achieved MAPQ ≥ 20. Supplementary alignment information, already encoded in the SA:Z tag of primary alignments, was used to classify read pairs as either split read pairs (containing supplementary alignments indicating intra-read breakpoints) or discordant read pairs (where mates mapped to different target gene loci without supplementary alignments). For split reads, breakpoint coordinates were determined by parsing CIGAR strings (Python ver. 3.10.12) from primary alignments to locate soft-clip boundaries, accounting for strand orientation. Fusion read pairs were counted by adding split read pairs and discordant read pairs. To establish the limit of detection (LoD), we incorporated triplicate NA12878 WGBS datasets as negative controls, which showed zero fusion reads, and applied probit regression analysis to calculate the fusion fraction detectable at the 95% confidence level.

## Supplementary Information

Supplementary Information 1.

Supplementary Information 2.

Supplementary Information 3.

## Data Availability

All raw FASTQ files are available from the NCBI Sequence Read Archive (SRA) database (BioProject accession number PRJNA1356354, https://www.ncbi.nlm.nih.gov/bioproject/PRJNA1356354). Previously published datasets (K562 WGS: SRR27705080; K562 WGBS: SRR4235743; MCF-7 WGBS: SRR8363862, SRR7707730; NA12878 WGS: SRR9091899; NA12878 WGBS: SRR20318443-SRR20318450, SRR10532136) are available from their original SRA accessions. All other relevant data are within the manuscript and its Supplementary Information files.

## Code availability

The code used in this study is available at https://github.com/kth-user/wgbs-fusion-detection.
