# Supplementary material for: Detection of known gene fusions in cancer cell lines using whole-genome bisulfite sequencing data
Source: Sci Rep. 2026 Mar 12;16:13254. doi: 10.1038/s41598-026-40803-0 (PMC13103421; doi:10.1038/s41598-026-40803-0)

**Supplementary information**

**Fig. S1 Distance of K562 breakpoints in WGBS compared to WGS.** Distribution of distances between breakpoints detected by WGBS and their nearest corresponding breakpoints were identified by WGS. For each WGBS-detected breakpoint, the closest WGS breakpoint was determined by minimal Euclidean distance using coordinates defined by breakpoint positions in *BCR* and *ABL1*. Distances are shown separately for *BCR* (**A**) and *ABL1* (**B**).

**
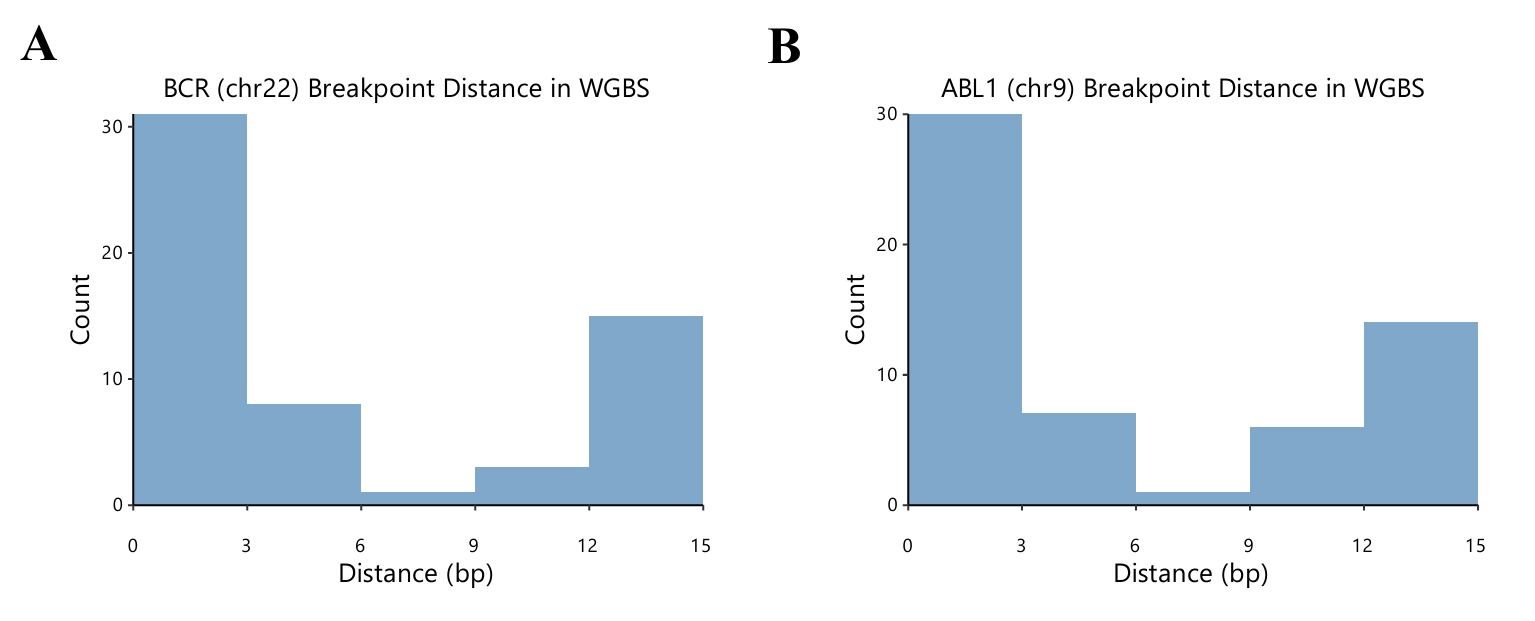
**

**Fig. S2 Coverage profiles of the *ABL1* gene surrounding the *BCR-ABL1* fusion breakpoint.** Gray shaded areas indicate the coordinate ranges where *BCR-ABL1* breakpoints were detected (top: WGS; bottom: WGBS).


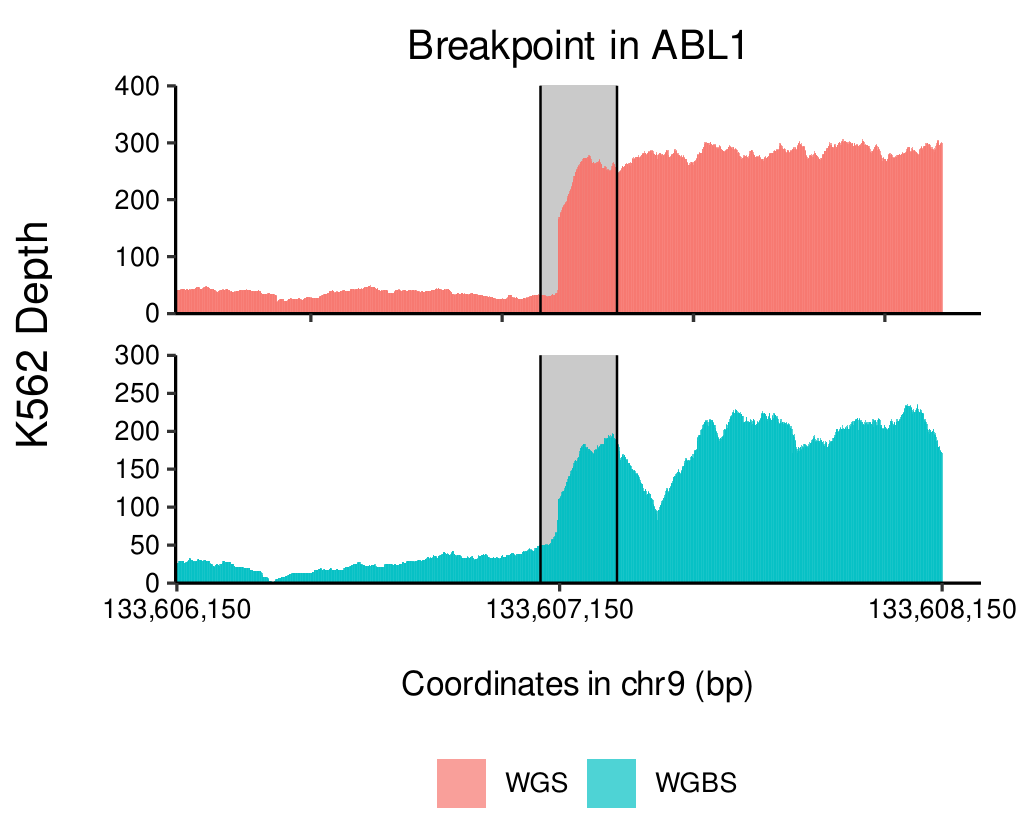


**Fig. S3 Methylation profiles of *BCR* gene surrounding the *BCR-ABL1* fusion breakpoint in WGBS.** Gray shaded areas indicate the genomic coordinate ranges where *BCR-ABL1* breakpoints were detected. Beta values, calculated as the fraction of methylated cytosines at individual CpG loci, are shown for K562 (*n* = 3) and NA12878 (*n* = 3).


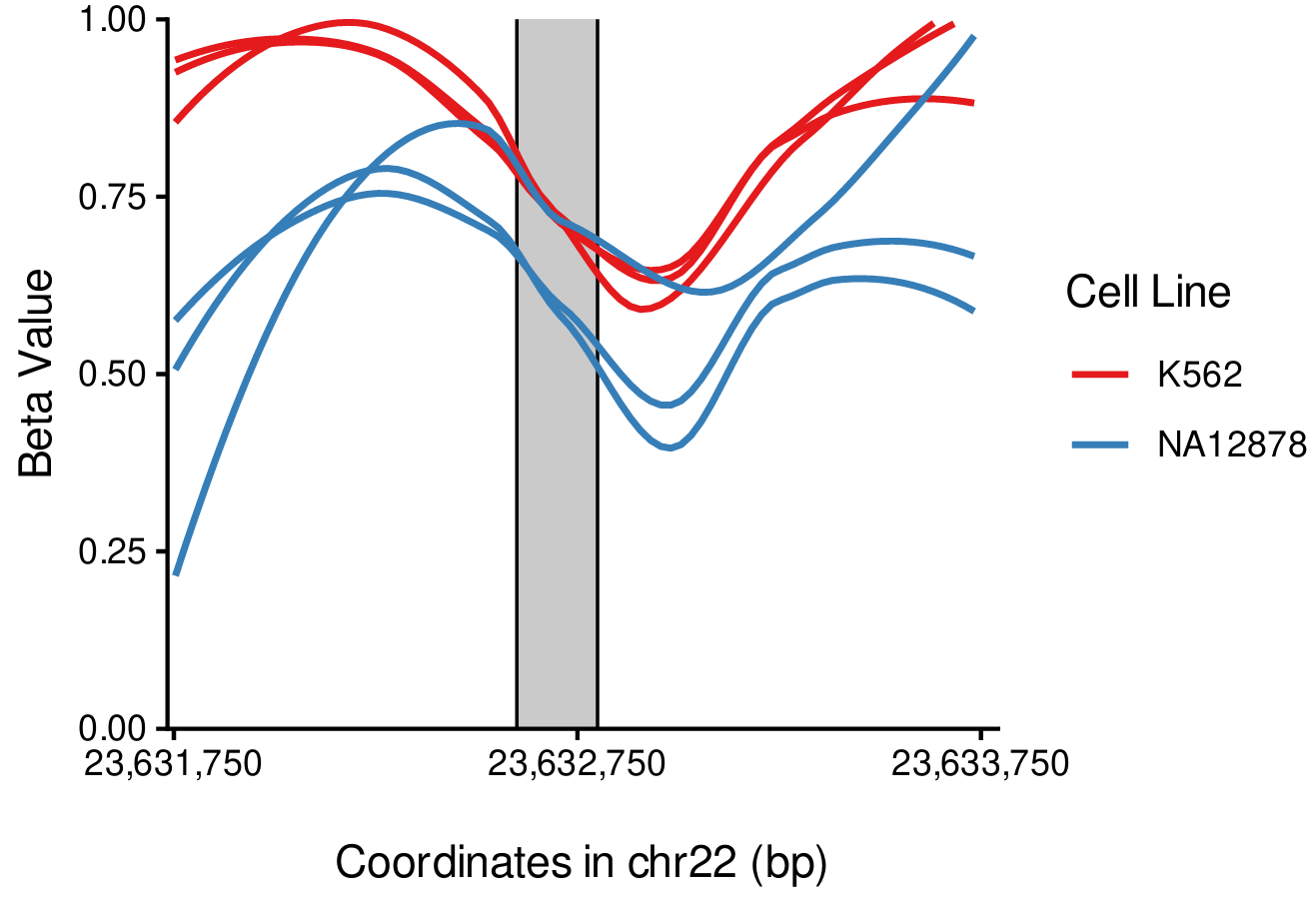


**Fig. S4 Downsampling analysis of K562 WGBS data.** The K562 WGBS sample was downsampled to various coverage depths (*n* = 10), and the number of unique fusion read pairs spanning *BCR* and *ABL1* was counted.


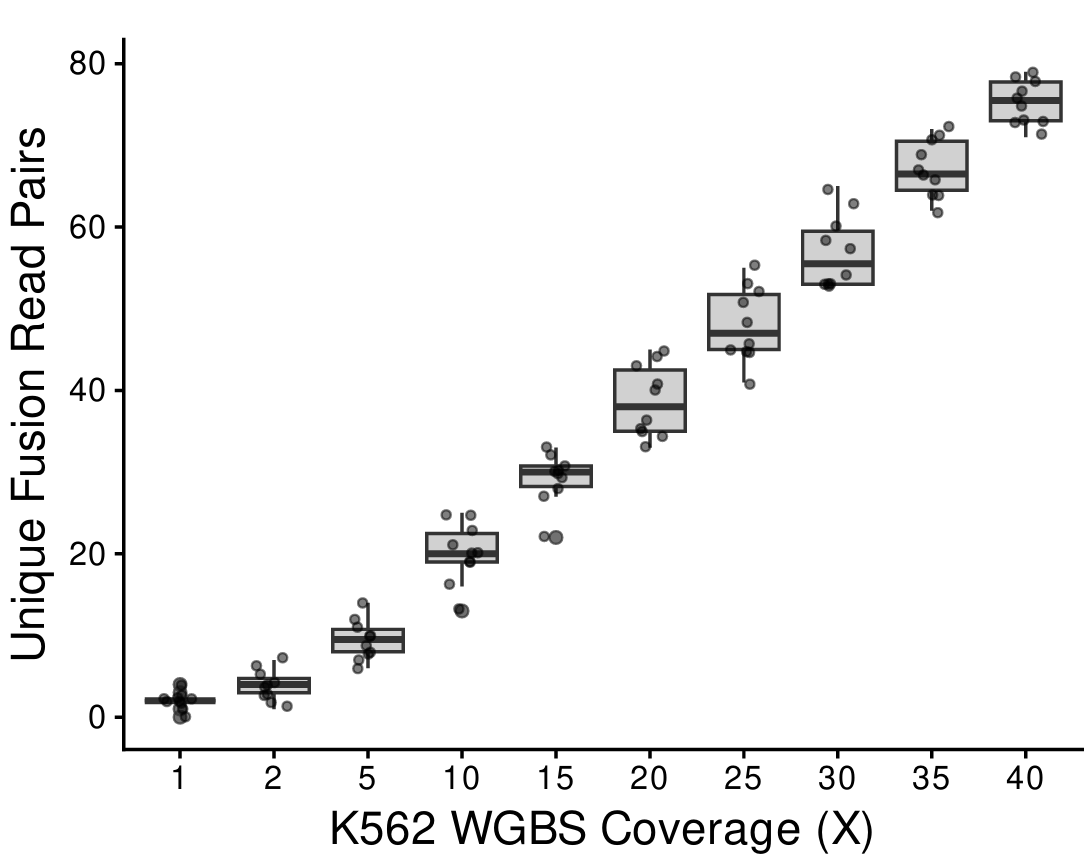


**Fig. S5 False discovery comparison between WGS and WGBS.** In NA12878 WGS and WGBS datasets, reads were downsampled to 5×, 10×, and 20× coverage, each in triplicate. The number of fusion read pairs detected across different genomic regions was assessed. “Whole-genome” refers to fusion events detected outside the target region, whereas “Target Region” refers to the single known fusion (*BCR-ABL1*). False discoveries in the whole genome and target region are shown in (**A**) and (**B**), respectively, with direct comparison between WGS and WGBS.


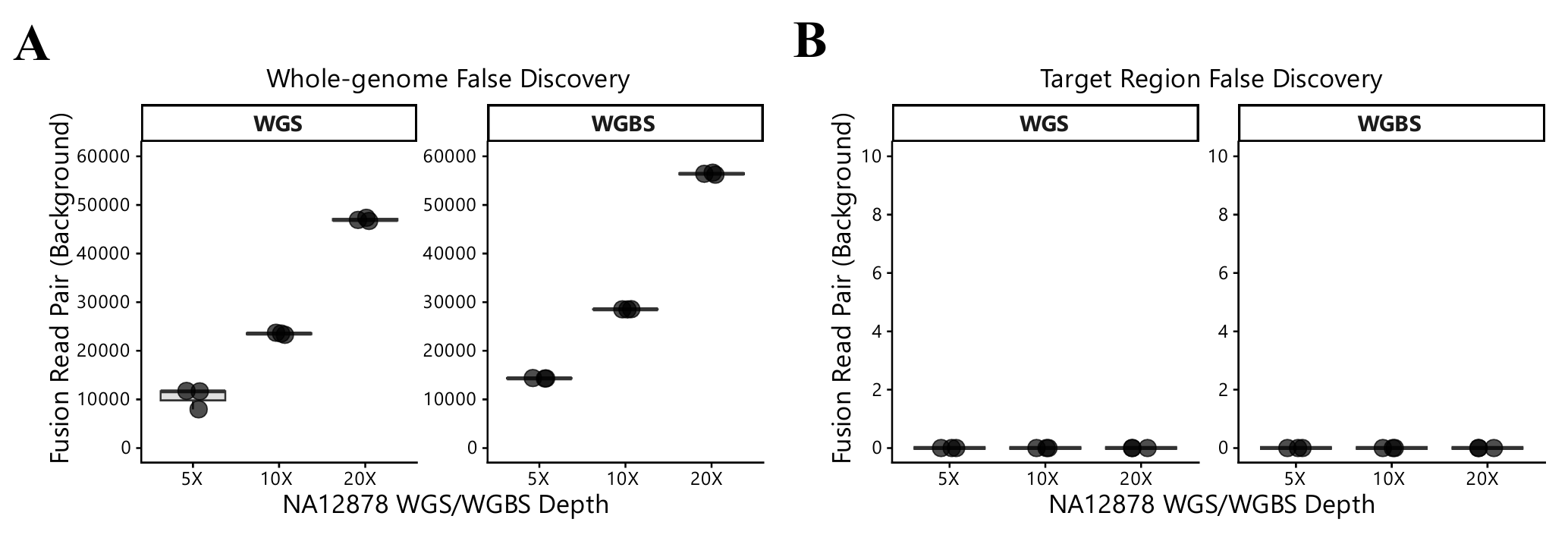


**Fig. S6 MAPQ distribution comparison for fusion candidate read pairs in WGS and WGBS.** Each MAPQ value represents the minimum mapping quality within the read pairs mapping to different genes. WGS and WGBS data from NA12878 (fusion-negative control) were downsampled to 20× depth. All detected fusion candidates represent false positives, with higher counts in WGBS indicating higher false positive detections.


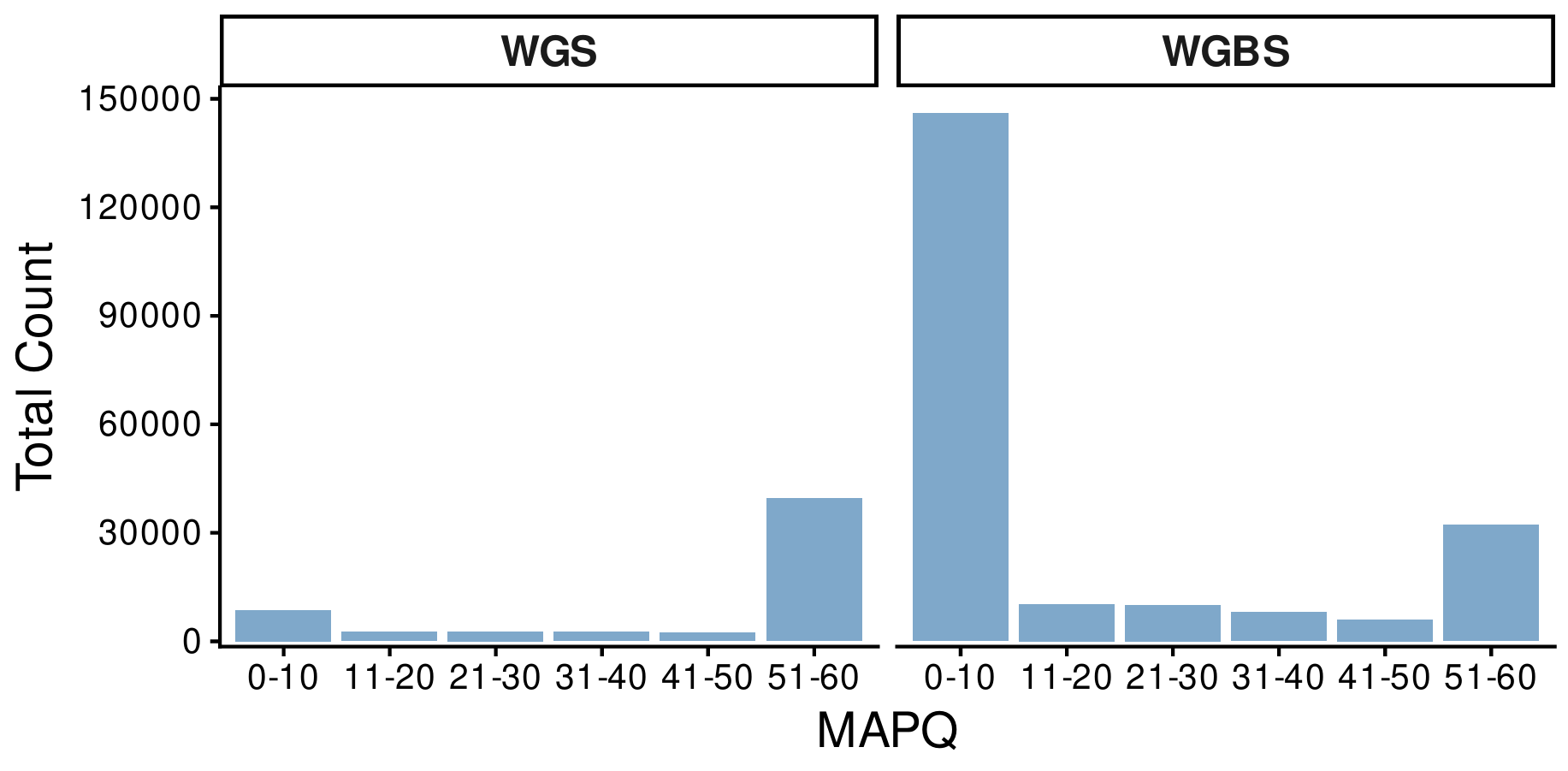


**Fig. S7 The number of false positives and true positives in fusion-positive cell lines.** False positives were defined as fusion read pairs detected outside the target region across the whole genome, while true positives were fusion read pairs detected within the target region. Fusion-positive cell lines K562 (**A**) and MCF-7 (**B**) were assessed.


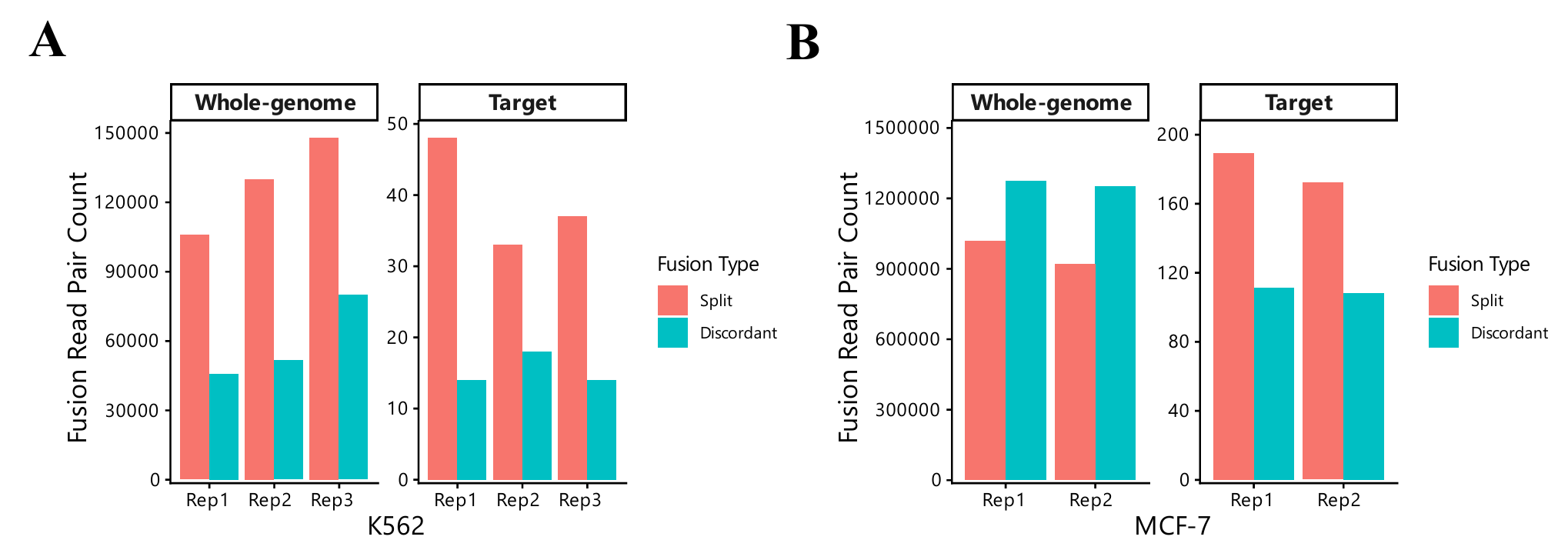

Supplement: Supplementary file 3 — Supplementary Information 3. [file 41598_2026_40803_MOESM3_ESM.docx]
